# Effect of histidine and carnosine on haemoglobin recovery in anaemia induced-kidney damage and iron-loading mouse models

**DOI:** 10.1007/s00726-025-03451-8

**Published:** 2025-05-12

**Authors:** Mayra Vera-Aviles, Jorge Moreno-Fernandez, Tugba Kose, Robert Hider, Gladys O. Latunde-Dada

**Affiliations:** 1 Department of Nutritional Sciences, School of Life Course and Population Sciences, Faculty of Life Sciences & Medicine, King’s College London, London, UK; 2 Department of Physiology, Anatomy and Genetics, University of Oxford, Oxford, UK; 3 Department of Physiology, Faculty of Pharmacy, Campus Universitario de Cartuja University of Granada, Granada, 18071 Spain; 4 Institute of Nutrition and Food Technology “José Mataix Verdú”, Biomedical Research Centre, University of Granada, Armilla, 18016 Spain; 5 Instituto de Investigación Biosanitaria (IBS) (E15-EXPODIET), Granada, 18016 Spain; 6 Department of Nutrition and Dietetics, Hitit University, Çorum, 19030 Türkiye; 7 Institute of Pharmaceutical Science, School of Cancer & Pharmaceutical Sciences, Faculty of Life Sciences & Medicine, King’s College London, London, UK

**Keywords:** Histidine, Carnosine, Adenine, Anaemia, Kidney, Disease

## Abstract

Histidine and carnosine can form complexes with divalent metal ions such as Fe^2+^, potentially providing stability to intracellular labile iron. Anaemia is a common comorbidity in the late stages of kidney disease, and patients are treated with erythropoiesis-stimulating agents (ESAs) and iron supplementation. However, iron supplementation is also associated with worse long-term outcomes. The purpose of this study is to investigate how histidine and carnosine supplementation can reduce symptoms of anaemia of chronic kidney disease (CKD) and the effects associated with iron-overloaded conditions. Adenine-induced chronic kidney disease mice were treated with histidine and carnosine by oral gavage for 10 days. Additionally, a model involving iron overload in mice was established, and these mice received concurrent treatment with histidine and carnosine. Haemoglobin, non-haem iron, malondialdehyde (MDA) and iron parameters were measured. Carnosine increased erythropoietin (EPO) levels (35.62 µg/ml ± 11.43) and resulted in haemoglobin repletion (16.7 g/dL ± 3.4). When iron was supplemented alongside with histidine or carnosine, there were better effects on haemoglobin repletion (14.22 ± 1.7 and 13.82 ± 2.15 g/ dL respectively), ferritin (59.5 ± 16.4, 52 ± 29.5 µg/ml) and non-haem iron (0.8 ± 0.21, 0.7 ± 0.38 nmol/mg), than the group receiving iron alone (*p* < 0.05). Furthermore, histidine and carnosine reduced non-haem iron and MDA, in iron-loaded conditions (*p* < 0.05). These positive effects observed in histidine and carnosine could be associated with reactive oxygen species (ROS) scavenging. EPO restoring levels in CKD model and the increment in haemoglobin and ferritin in carnosine treatments suggested the potential formation of a ternary complex with iron-glutathione. In conclusion, our results indicate the beneficial effect of histidine and carnosine in the context of iron supplementation for the correction of haemoglobin and protection against iron-loaded conditions.

## Introduction

During Chronic Kidney Disease (CKD), especially in late stages, inflammation is associated with dysfunctional erythropoiesis and elevated levels of hepcidin, the hormone that master regulates iron body homeostasis (Babar and Saboor 2024). The current treatment for iron deficiency anaemia in CKD is based on erythropoiesis-stimulating agents (ESAs) along with iron supplementation (Wong et al. 2019; Batchelor et al. 2020). However, high hepcidin levels impair iron metabolism and the response to iron supplementation (Atkinson and White 2012). Furthermore, even though the current intravenous iron formulations are claimed to be safer, there still is an increase in oxidative stress after supplementation dissociated with the release of NTBI (Espósito et al. 2002; Prakash et al. 2005; Lee et al. 2006). A better approach to current therapies is needed for the management of iron deficiency anaemia in patients with CKD.

The use of antioxidants as part of CKD treatment has been considered in recent years (Ikizler et al. 2020). Research has shown that vitamin C supplementation is associated with improved outcomes for haemoglobin (Hb) levels. When administered in smaller doses (300 mg three times a week), ascorbic acid increases the availability of iron for erythropoiesis and helps enhance the correction of erythropoietin (EPO) levels (Altemose et al. 2018; Kędzierska-Kapuza et al. 2023). However, the advantages of daily vitamin C supplementation remain uncertain, particularly for patients receiving iron supplements, as ascorbic acid can also have pro-oxidant effects (Conner et al. 2012). Additionally, correcting vitamin D levels has been linked to improved haemoglobin and EPO responses (Icardi et al. 2013; Altemose et al. 2018), Nevertheless, the long-term benefits of this correction remain unclear for patients with iron deficiency anaemia and those in the later stages of CKD (Wang et al. 2023a, b). Histidine and the histidine dipeptide carnosine (β-alanine-L-histidine), have been studied as supplements during exercise and in chronic inflammatory conditions due to their buffering and ROS quenching capacity (Blumenkrantz et al. 1975; Kopple and Swendseid 1975; Lamont and Miller 1992; Vera-Aviles et al. 2018; Peters et al. 2019). Supplementation with histidine and carnosine was revealed to have beneficial effects in reducing inflammatory markers in diabetes and kidney disease (Peters et al. 2015, 2019; Menon et al. 2018). Both, histidine and carnosine, are naturally present in the plasma and tissues under physiological conditions (Watanabe et al. 2008; Kilis-Pstrusinska 2020).

Several studies reported the ability of histidine and carnosine to form complexes with metal ions, such as Fe^2+^, Cu^2+^, Co^2+^, Ni^2+^, Cd^2+^, and Zn^2+^ (Lenz and Martell 1964). Specifically, histidine is responsible for binding iron in haemoglobin and myoglobin molecules and is frequently present in the active sites of metalloenzymes (Williams 1970; Wade and Tucker 1998; Iyengar et al. 2010; Kang 2010; Zhang et al. 2016). Both histidine and carnosine have the potential to form complexes with intracellular labile iron, proving better stability and making iron more bioavailable for use and transport to organelles such as mitochondria (Aruoma et al. 1989; Mannion et al. 1995; Baguet et al. 2011; Varanoske et al. 2017; Baye et al. 2019; Hider et al. 2021). In clinical studies, the administration of carnosine in patients with chronic heart failure resulted in significant improvement in exercise capacity, assessed by cardiopulmonary exercise testing, without changes in left ventricular ejection fraction compared with untreated control patients (Lombardi et al. 2015).

In this study, we present evidence that supplementing histidine and carnosine can counteract the excessive production of ROS and reduce tissue damage associated with iron loading. Moreover, the findings indicate that this supplementation plays a role in correcting anaemia in chronic kidney disease (CKD) and iron-overload related conditions.

## Methods

### Study design of animal experimentation studies

#### Adenine-induced anaemia of CKD

Twenty BALB/c male mice, four weeks old were housed at 21–23⁰C in a 12-hour dark/light cycle. The animals were fed with a standard laboratory pellet diet (TD 80394, 48 ppm iron, ENVIGO, Indiana, US) and water ad libitum. The adenine treatment (50 mg/kg weight) (cat. A8626, Sigma-Aldrich, Germany) was given by oral gavage for 4 weeks using 0.5% carboxymaltose (CMC) as a vehicle (Hanudel et al. 2017; Rahman et al. 2018). A group, namely control untreated group (group 1) (*n* = 8 mice) was treated only with the vehicle (0.5% CMC) for the same time length. Following adenine treatment, haemoglobin levels and urea nitrogen in plasma (BUN) were measured to confirm kidney damage and anaemia. Mice with adenine-induced kidney damage were divided into 6 treatment groups: (1) Water, (2) Histidine, (3) Carnosine, (4) Iron, supplementation with ferric carboxymaltose (FCM) (5) FCM and histidine, and (6) FCM and carnosine. Histidine and carnosine were provided in a dose of 1 g/L for four weeks, the dose was chosen from previous studies that showed a protective effect against oxidation and glycation (Lee et al. 2005). Three groups of mice (groups 4–6) were treated with ferric-carboxymaltose (FCM) by intraperitoneal injection (i.p.) in a dose recommended for the guidelines to treat anaemia in CKD (NICE Guideline and National Clinical Guideline Centre: London, 2015; Ratcliffe et al. 2016) and proportional to the body weight of the mice (0.5 mg iron /100 µl saline solution), one injection per week for two consecutive weeks (week 7 and 8). All in-vivo experiments were carried out under Home Office approved conditions and animal care, the regulation of scientific procedures met the criteria laid down by the United Kingdom Animals (Scientific Procedures) Act 1986.

#### Iron-overload

Iron supplementation is used to treat anaemia in CKD, and it is commonly associated with oxidative stress and iron-related toxicity in tissues; for this reason, a model of iron-overload was used to test the efficiency of histidine or carnosine to protect tissues against iron-induced oxidative stress. Four-week-old mice were divided randomly into four groups (*n* = 5): (1) Control, (2) Iron-loaded with iron dextran (ID), (3) Histidine + iron-loaded, and (4) Carnosine + iron-loaded. Iron dextran was administered in five intraperitoneally (i.p.) doses injected (100 mg iron/kg mouse body weight) every two days for 10 days to induce the iron overload condition. Mice were given histidine or carnosine (100 µL of 1 g/L histidine or carnosine) for 14 days by oral gavage a day before starting iron dextran treatment. The control untreated mice were given water by gavage for 14 days. Euthanasia of the mice was performed on day 15 by injecting 0.4–0.6 mL of pentobarbitone sodium 20% w/v solution (i.p.) to the mice.

#### Haemoglobin quantification

Blood was withdrawn from the tails of the mice to determine the baseline haemoglobin (Hb) levels after the adenine treatment (day 28) for the CKD model and at the end of carnosine/histidine treatment in both experimental models. Hb determination was performed by adding 5 µl of whole blood to 995 µl of Drabkin’s reagent (1:200) (cat. D5941,Sigma-Aldrich, Germany) and measuring the optical density at 540 nm (Kajarabille et al. 2017).

#### Biochemical analysis

Blood urea nitrogen (BUN) was detected using a commercial kit (cat. EIABUN, Thermo Fisher, London, UK). ELISA ferritin assay was performed with a kit supplied by Abcam (cat. ab157713, Abcam, Cambridge, UK), and plasma samples were diluted 1:40 with the assay diluent provided in the kit. Total glutathione (GSH) in the liver was measured with an ELISA kit (cat. MBS026635, MyBiosource, Sand Diego CA, USA) and EPO concentration in the kidney using Abcam ELISA kit (cat. ab119593, Abcam, Cambridge, UK).

#### Tissue sample Preparation

All Tissue samples were rinsed in ice-cold PBS (pH 7.4) to remove excess blood thoroughly, blot-dry in tissue paper and snap-freeze in liquid nitrogen. Before analysis, tissues were crushed using liquid nitrogen and weighed. For EPO ELISA, kidney tissue powder (20 mg) was minced and homogenized in a glass Dounce homogenizer with 200 µL PBS on wet-ice and centrifuged at 1,500 g for 10 min to collect the supernatants which were used undiluted. For total GSH liver (20 mg) samples were homogenized in 1 mL glass Dounce homogenizer with 10 volumes of cold 5% Sulfosalicylic acid (SSA) to deproteinize the sample. After 10 min of incubation on ice, the samples were centrifuged at 10.000 g for 10 min at 4 °C and the supernatant was used for the determination of total glutathione.

#### Hepcidin

Hepcidin was determined in plasma samples according to the method reported by Bansal et al. 2010. Using a mass spectrometry with TSQ Quantum Access triple quadrupole and the LTQ-XL linear ion trap (Thermo, San Jose, CA, USA). Optimization was performed by infusion of mouse hepcidin standards and extrapolating the values of the standard curve values for mouse hepcidin.

#### Lipid peroxidation (MDA)

Lipid peroxidation was measured by the detection of the endpoint product malondialdehyde (MDA), using the MDA microplate assay kit from Cohesion Biosciences (cat. CAK1011, Cohesion Bioscience, London, UK). The absorbance was read at 532 and at 600 nm using a plate reader. The results were normalized against protein content of the samples. To measure the protein content, the samples were diluted 1:10 with the MDA assay buffer using the Bradford method.

#### Non-haem iron determination in mouse tissues

Non-haem iron was evaluated according to the modified method of Simpson and Peters (1990). Briefly, protein precipitation is performed with trichloroacetic acid (TCA). Ferric iron is released with sodium acetate solution (pH 4.8), which is reduced to ferrous iron Fe(II) by sodium ascorbate which then reacts with sodium 3-(2-pyridyl)-5,6-bis(4 phenylsulfonate)-1,2,4-triazine (ferrozine) to form a purple Fe-ferrozine complex that can be read spectrophotometrically (Simpson and Peters 1990). Crushed tissue samples of liver, kidney, and spleen were homogenized in 500 µL of 0.15 M NaCl in 10 mM NaOH-HEPES buffer (pH 7.4) in a 1 mL glass Dounce homogenizer. In a screw cap tube, 100 µL of the homogenate was mixed with 200 µL of extraction buffer mixture (25% TCA in 4% sodium pyrophosphate). The tube was placed in a boiling water bath for 10 min, centrifuged for 5 min at 14,500 g and the supernatant was collected in a new Eppendorf tube. The precipitate was re-suspended with 100 µL of TCA, heated and further centrifuged. After a third extraction, 200 µL of the overall extract was mixed with 100 µL of 0.23  M ascorbic acid, 80 µL of 0.01 M ferrozine and 420 µL of 2.0 M sodium acetate buffer (pH 4.8). The blank and the standard solutions were prepared similarly, except that 200 µL of TCA was added to each tube. As a standard, 1.0 µL of 10 mM ferric chloride [FeCl_3_ dissolved in 10 mM hydrochloric acid (HCl)] was used. The optical densities of all tubes were measured at 562 nm against the blank using a Camspec M330 UV-Visible spectrophotometer. All reagents were purchased from Sigma Aldrich (Sigma-Aldrich, Germany).

## Results

### Effect of histidine or carnosine in anaemia of CKD

**Fig. 1:**
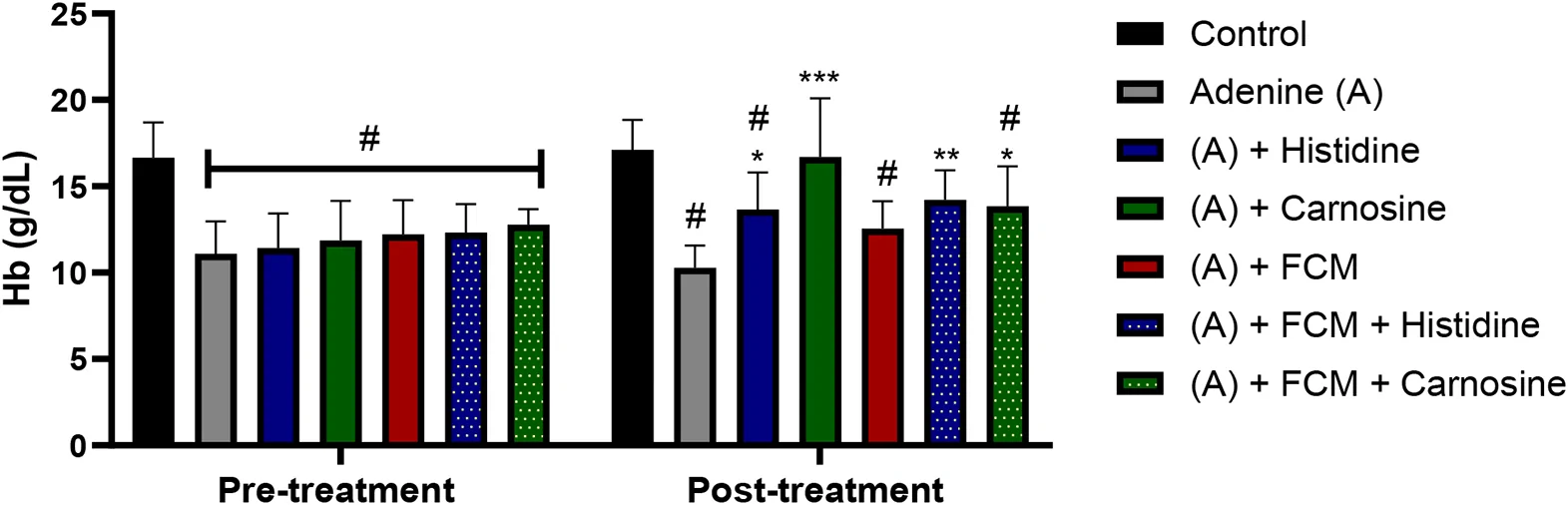
Haemoglobin levels pre and post-treatment with histidine or carnosine. All the groups were compared at the baseline to observe differences with the control group. * significantly different (p ˂ 0.05) from the control group. * (p ˂ 0.05) **(p ˂ 0.005) ***(p ˂ 0.001) significant difference between the adenine group and the experimental treatments. Two-way ANOVA. Sample size *n* = 8. Ferric carboxymaltose (FCM)

After adenine treatment, baseline haemoglobin (Hb) values were significantly different between the control group (16.66 ± 1.71 g/dL) and all the adenine-treated groups (11.75 ± 2.8 g/dL) (*p* < 0.05). The treatment of adenine-induced anaemia of CKD in mice with histidine or carnosine (1 g/L in drinking water) for 4 weeks demonstrated a positive effect on the Hb repletion (Fig. 1). Histidine and carnosine, with and without iron supplementation, significantly enhanced Hb levels compared to the adenine-induced kidney damage treated group (histidine 13.65 ± 2.13 g/dL, carnosine 16.69 ± 3.4 g/dL, and adenine 10.27 ± 1.3 g/dL). Whereas intravenous iron supplementation alone (12.54 ± 1.59 g/dL) did not significantly increase Hb levels. Carnosine treatment alone showed the highest Hb repletion levels (p ˂ 0.001), presenting a recovery of Hb levels comparable with the control group (Fig. 1).

**Fig. 2:**
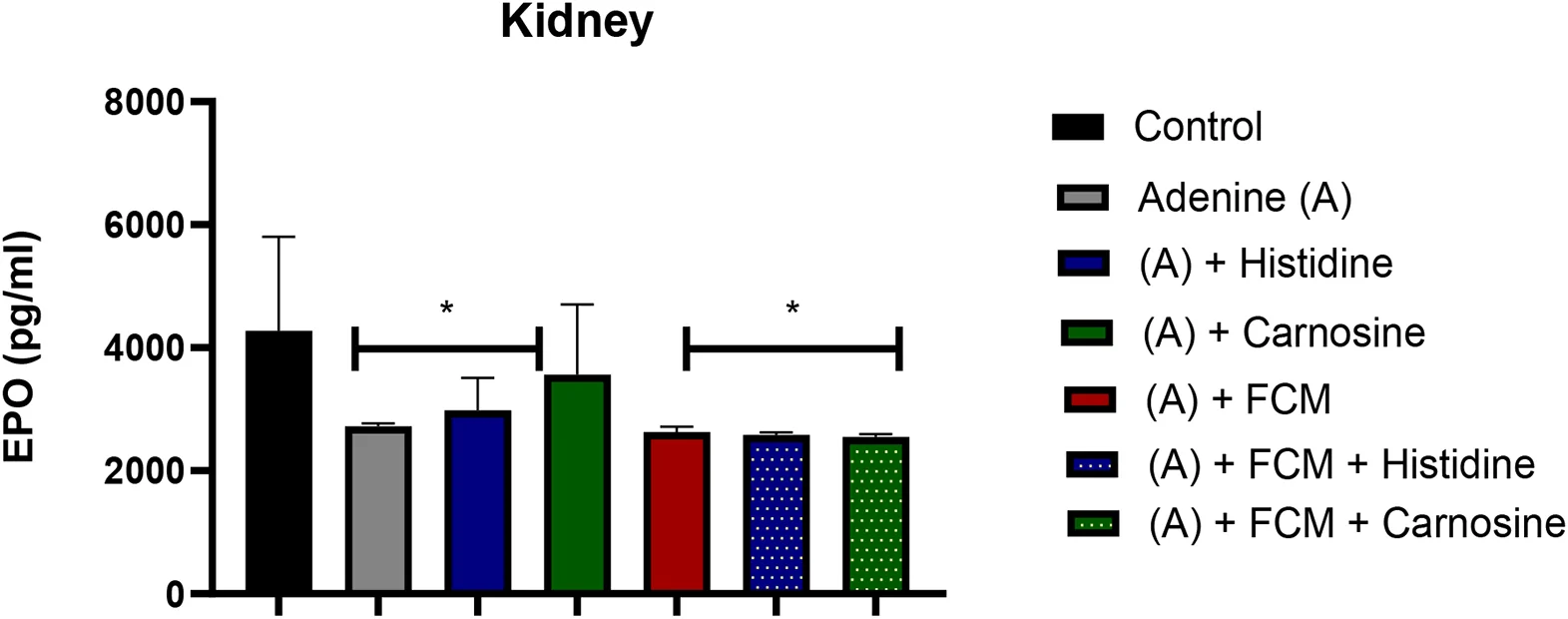
Erythropoietin (EPO) levels in kidney tissue. Carnosine treatment ((A) + Carnosine) presented levels similar to the control untreated group. * treatment significantly different from the control group (p ˂ 0.05). ANOVA one-way test. sample size *n* = 8. Ferric carboxymaltose (FCM)

Erythropoietin (EPO), the hormone that induces erythrocyte synthesis, was evaluated in the kidney to assess recovery of tissue damage. The treatment with carnosine alone increased EPO levels comparable to normal values (Fig. 2). All the other treatments did not significantly influence EPO levels in the kidney.

**Fig. 3:**
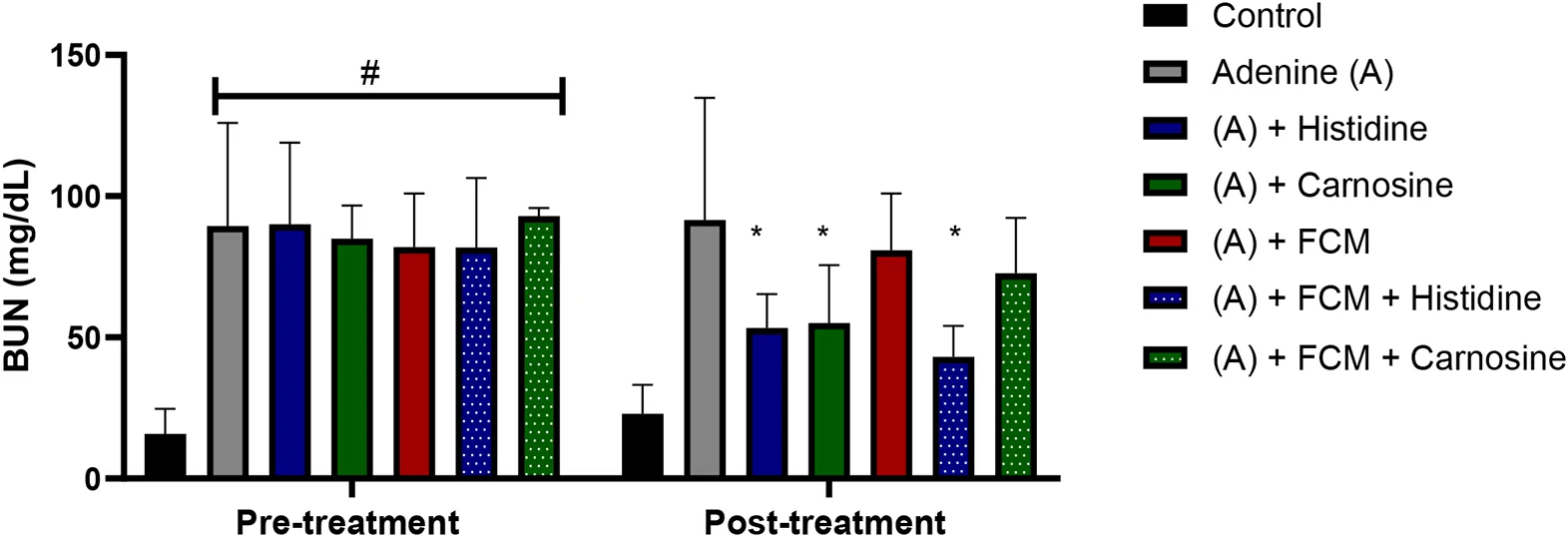
Blood urea nitrogen (BUN) levels in plasma. Urea nitrogen levels were measured pre and post-treatment. All the groups treated with adenine presented similar levels of BUN at the baseline and were significantly different from the control untreated group (# p ˂ 0.05). After treatment with histidine or carnosine, BUN levels were decreased significantly. # significant difference (p ˂ 0.05) between the control and treatment groups. * significant difference (p ˂ 0.05) between the adenine group and the other treatments. ANOVA two-way test. Sample size *n* = 8. Ferric carboximaltose (FCM)

**Fig. 4:**
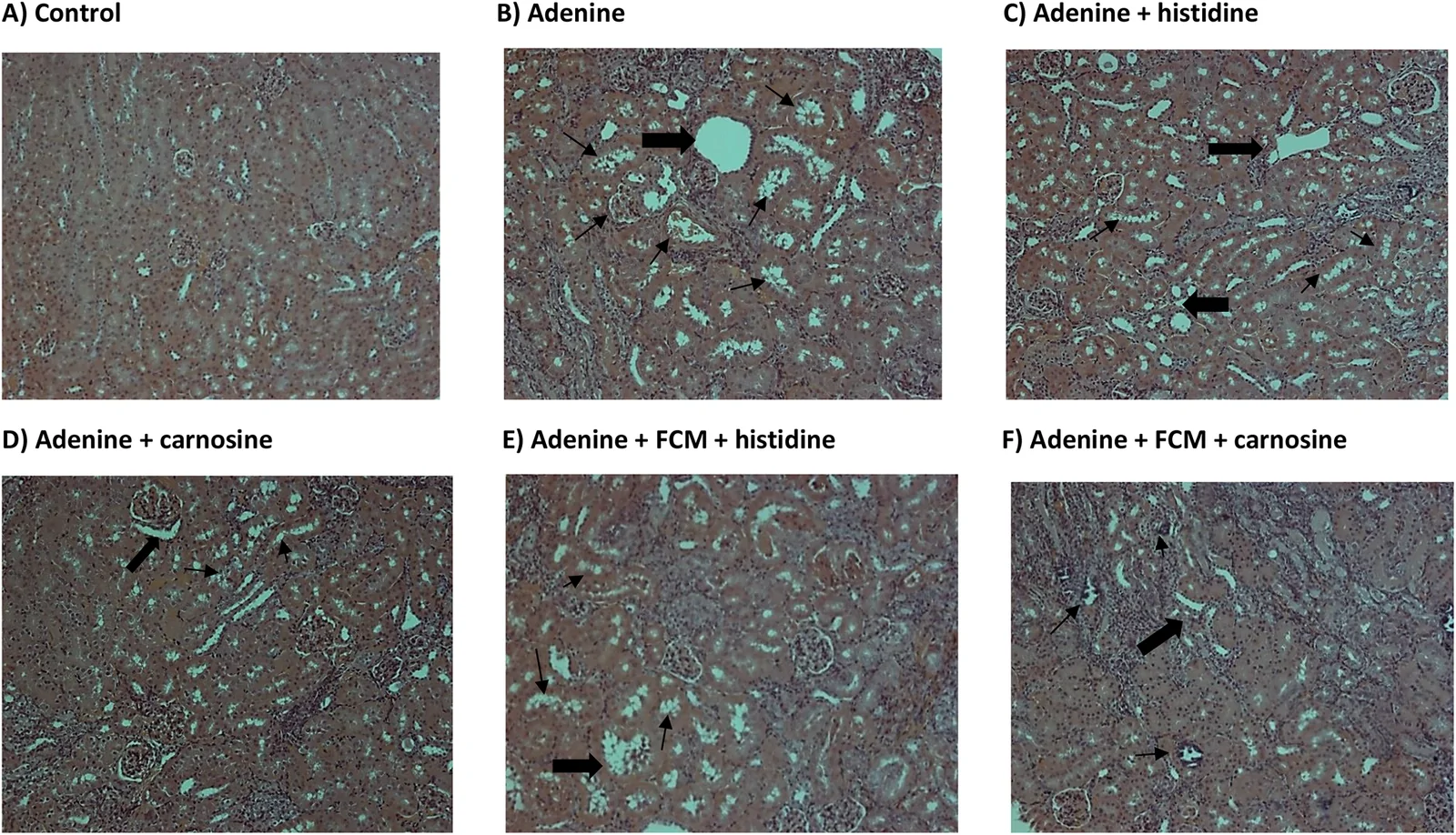
Haematoxylin and eosin (H & E) staining of kidney tissues. Black arrows show alteration in tubular morphology, and thinner arrows indicate crystal accumulation and necrotic cellular debris. (*n* = 2 slides)

**Fig. 5:**
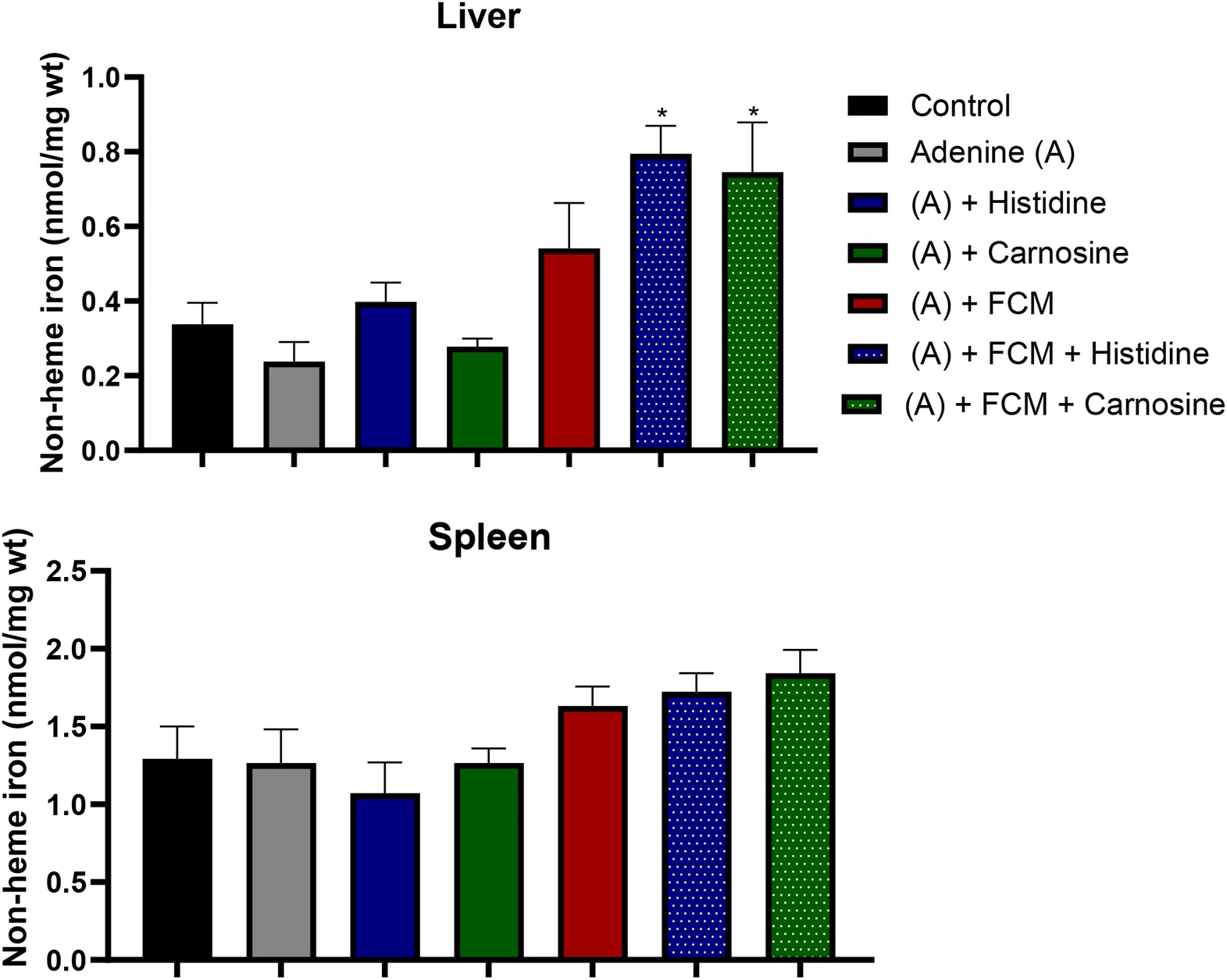
Non-haem iron levels in the liver (**A**) and spleen (**B**). Significant differences were found in the liver of the group supplemented with Ferric carboxymaltose (FCM) in combination with carnosine (* p ˂ 0.05). ANOVA two-way test. Sample size *n* = 8

Urea nitrogen in blood (BUN) is a marker of kidney damage, and higher levels in plasma indicate a lower glomerular filtrate rate. Treatment with histidine and carnosine demonstrated an amelioration in the kidney damage, decreasing BUN levels, furthermore, amongst the iron-supplemented groups, histidine decreased BUN levels significantly (p ˂ 0.05) in comparison with the group treated with adenine or FCM alone (Fig. 3). Changes in kidney tissue caused by adenine-induced kidney damage presented tubular dilation, crystal accumulation, and cast formation with necrotic cellular debris as detected by haematoxylin and eosin staining (Fig. 4). Histidine and carnosine treatments showed an amelioration in tissue damage, with fewer alterations in the glomerular structure. However, tubular alterations were still evident. To understand the effects of histidine or carnosine on iron deposition in tissues, non-haem iron levels in the liver and spleen were determined (Fig. 5). In the liver, all three groups supplemented with intravenous iron presented higher non-haem iron levels; however, when groups were treated with histidine or carnosine, significantly higher levels were observed in both tissues in comparison with the group treated with iron supplementation alone (p ˂ 0.05) (Fig. 5). These trends were in line with haemoglobin recovery presented previously (Fig. 1).

**Fig. 6:**
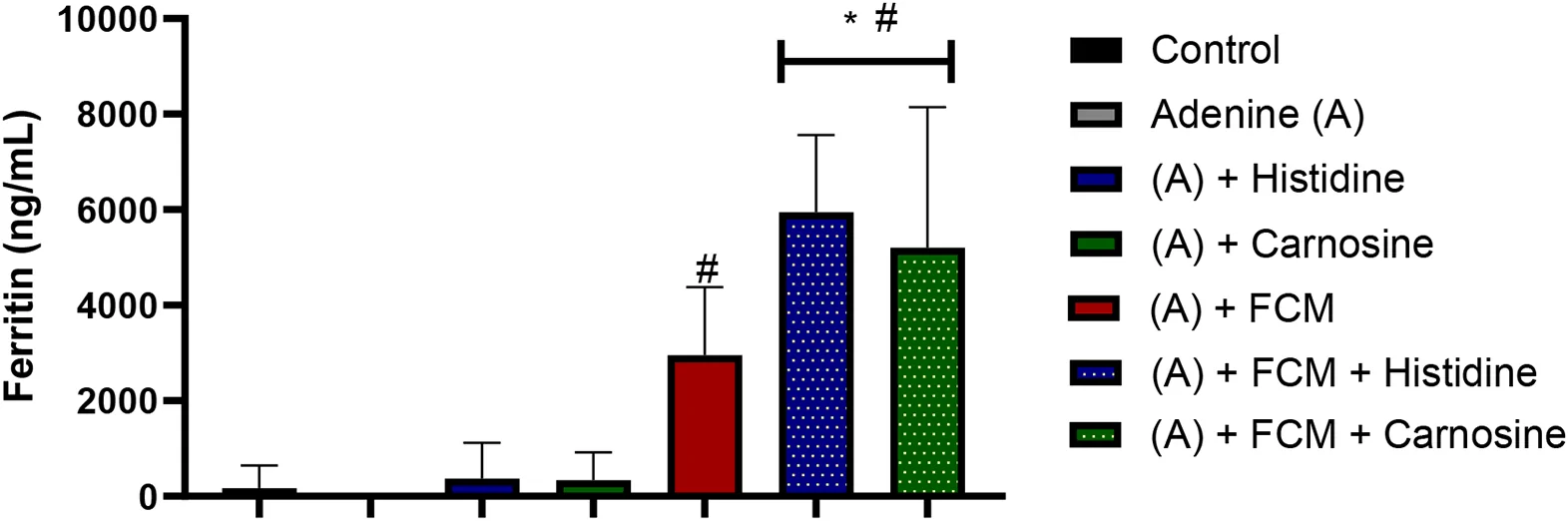
Ferritin levels in plasma. The groups with Ferric carboxymaltose (FCM) presented higher values than the control and the adenine group (# p ˂ 0.05). The groups with iron supplementation with Ferric FCM combined with histidine and carnosine showed higher values than those that received FCM alone (* p ˂ 0.05). ANOVA two-way test. Sample size *n* = 8

**Fig. 7:**
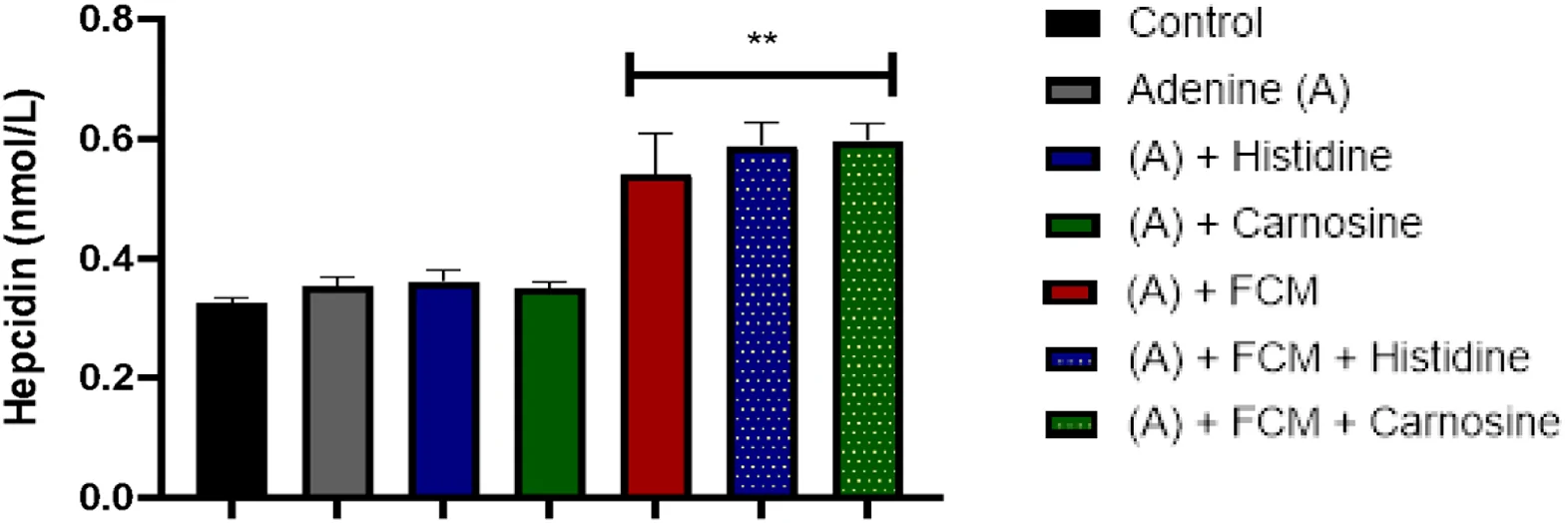
Hepcidin levels in plasma. Values presented in the bars are means ± SEM (*n* =8). The asterisk represents a significant difference between the control group (**p ˂ 0.005) and the treatments. Hepcidin levels were higher in the iron-supplemented groups with Ferric carboxymaltose (FCM) than in the control and adenine groups. ANOVA one-way test. Sample size *n* = 8

As expected, groups treated with intravenous iron presented higher hepcidin and ferritin levels in plasma. This is expected since hepcidin responds to systemic iron and is secreted when iron levels in plasma increase; ferritin is expected to increase in response to iron supplementation. Interestingly, the effect of histidine or carnosine treatment in combination with intravenous iron resulted in higher ferritin levels than supplementation of iron alone (p ˂ 0.05) (Figs. 6 and 7).

Our data shows that histidine and carnosine treatments positively influence the recovery of haemoglobin, suggesting an increase in iron bioavailability, particularly in combination with iron supplementation.

### Effect of histidine or carnosine in iron-loaded conditions

**Fig. 8:**
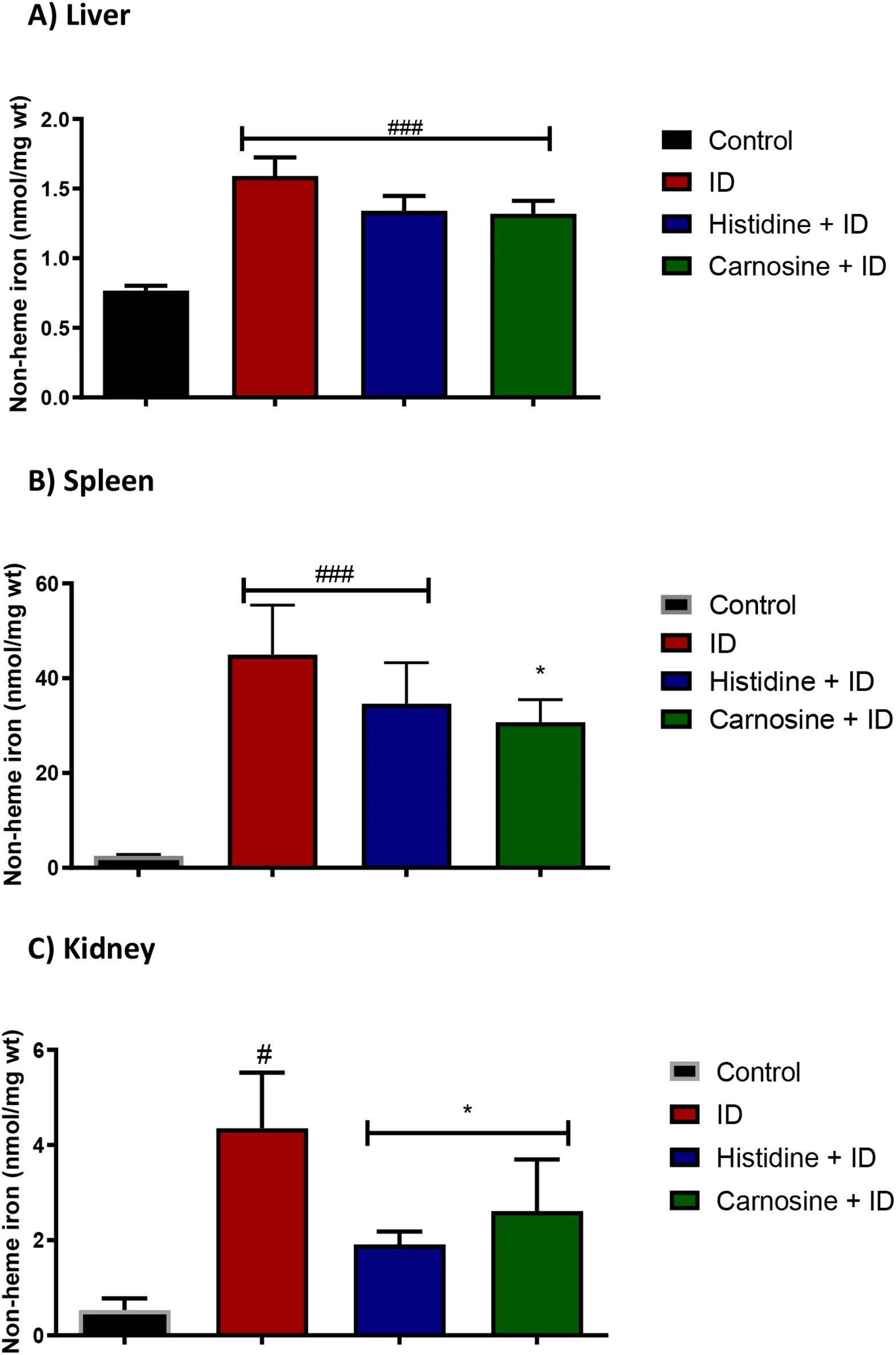
Non-haem iron levels in tissues. Figure (**A**) shows the non-haem levels in the liver, (**B**) spleen and (**C**) kidney. In the liver (**A**), all the groups presented higher levels of iron (*p*< 0.05) compared with the control group. Carnosine reduced non-haem levels in the spleen (**B**) and kidney (**C**).iron-loaded group with iron dextran (ID). # significantly different from the control group (p ˂ 0.05). * Significantly different from the iron dextran group (p ˂ 0.05) for ANOVA one-way test. Sample size *n* = 5

**Fig. 9:**
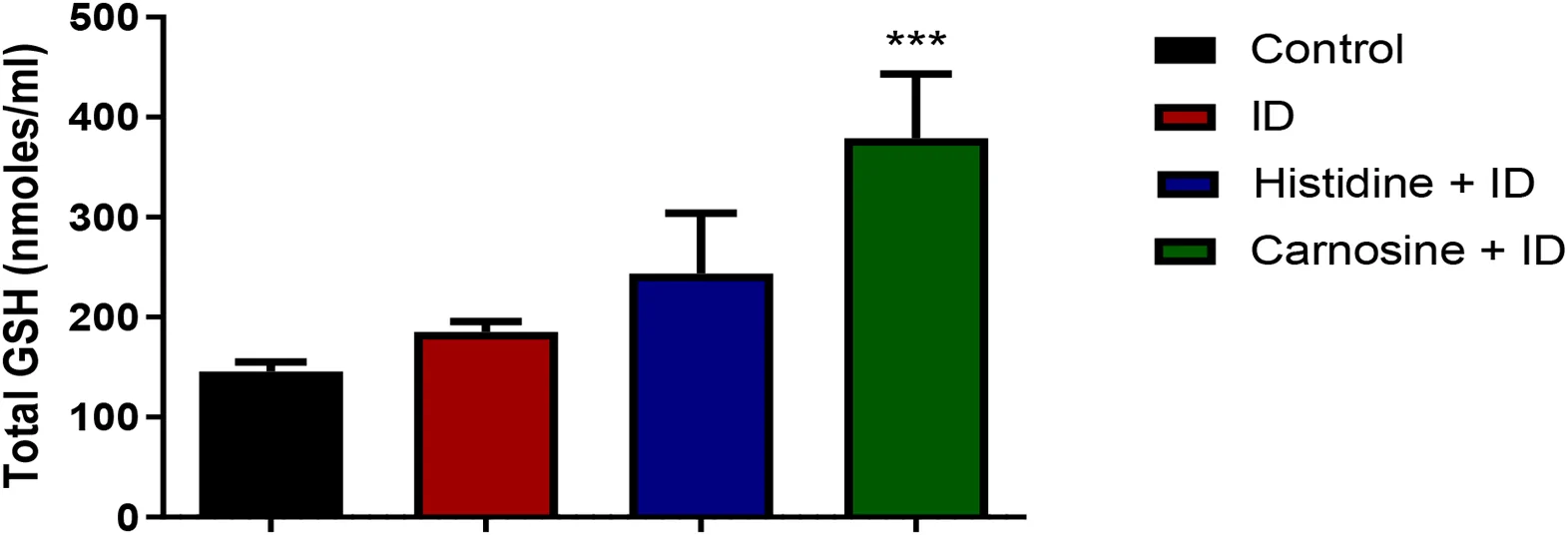
Glutathione levels in the liver. Histidine and carnosine groups presented higher (*p*< 0.05) levels of glutathione levels (GSH) in the liver compared with the control. Iron-loaded group with iron dextran (ID). *** Significantly different from the Fenton substrate (FS) group (p ˂ 0.001) for ANOVA one-way test. *n* = 5

**Fig. 10:**
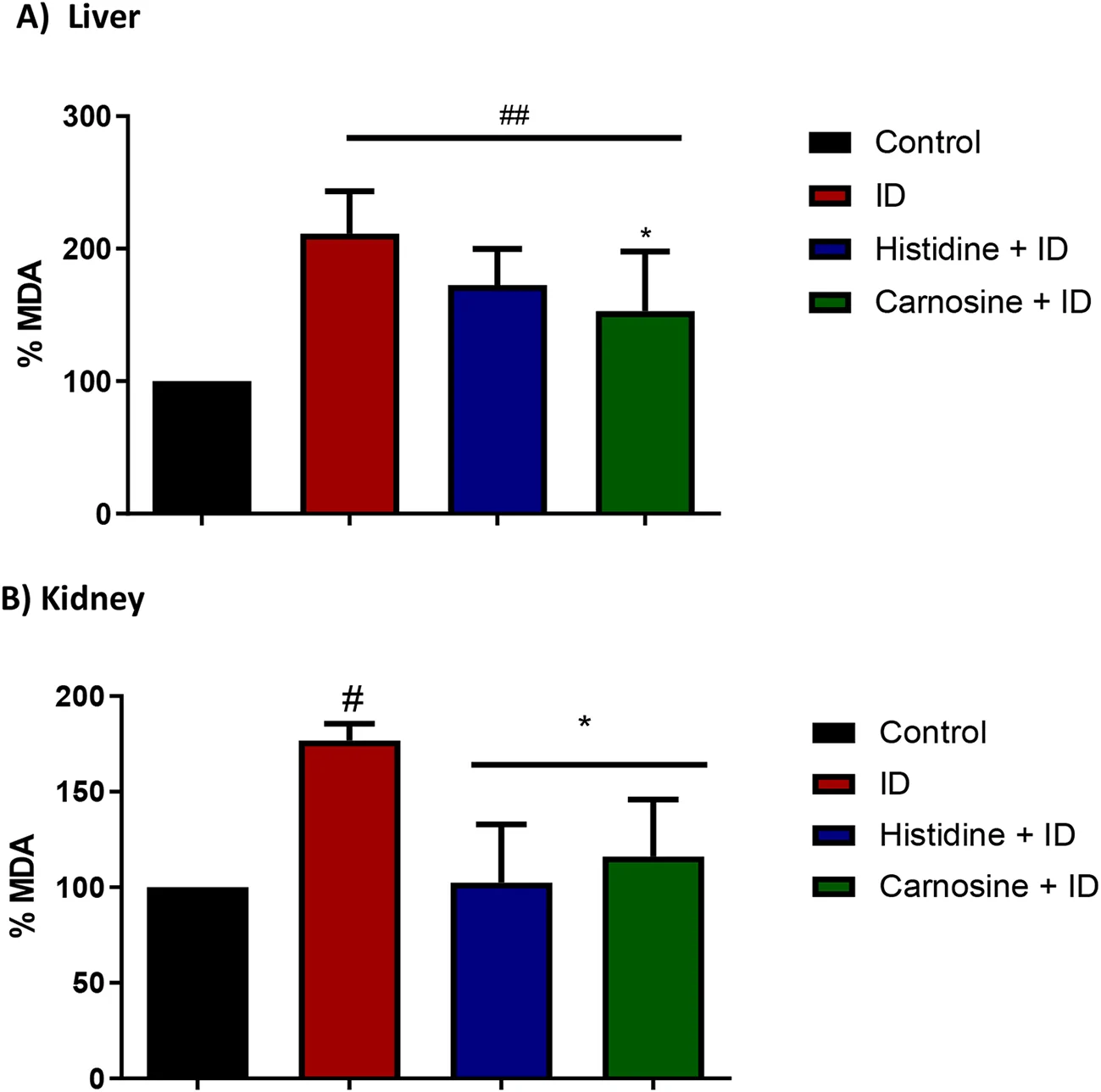
Malondialdehyde (MDA) assay in the liver (**A**) and kidney (**B**) tissue. In the liver (**A**) iron dextran (ID) and histidine groups showed higher levels of MDA (*p*< 0.05) compared with the control group in liver samples. In contrast, the carnosine group has lower levels of MDA, similar to the control group. In the kidney (**B**), histidine and carnosine groups presented lower levels of MDA, similar to the control group (*p*< 0.05). iron-loaded group with iron dextran (ID). Histidine and carnosine were supplemented at 1 g/L. ANOVA one-way, *n* = 5

We evaluated iron accumulation in the tissues using a non-haem iron assay to analyse whether histidine and carnosine influence iron deposition levels in the liver, spleen, and kidney. In the liver, the groups subjected to iron overload exhibited increased levels of non-haem iron, and the supplementation with histidine and carnosine did not change the iron deposition in liver tissue appreciably (Fig. 8-a). However, carnosine decreased the amount of non-haem iron accumulation in the spleen and kidney tissues (*p* < 0.05) (Fig. 8). Histidine treatment presented the same effect as carnosine only in the kidney tissue. In the liver, only carnosine treatment resulted in higher levels of GSH compared to the iron-loaded group (*p* < 0.001) (Fig. 9). To measure the effect on lipid peroxidation protection, the MDA assay in liver and kidney tissue was performed; carnosine decreased the levels of MDA in both tissues in comparison with the iron-loaded group (*p* < 0.05). Histidine significantly decreased (*p* < 0.05) MDA level only in the kidney sample (Fig. 10).

## Discussion

### Histidine and carnosine corrected Hb levels

The purpose of this study was to investigate the effects of histidine and carnosine on the restoration of two conditions: (1) anaemia of kidney disease induced by adenine and (2) tissue iron-overload in mice. The adenine model of anaemia of chronic kidney disease (CKD) induces damage by the accumulation of 2,8-dihydroxyadenine crystals in the proximal tubules of the nephron, leading to inflammation and tubulointerstitial fibrosis and anaemia (Rahman et al. 2018). Our results showed that histidine and carnosine increased haemoglobin (Hb) levels and reduced blood urea nitrogen (BUN) in plasma after 8 weeks of treatment in drinking water (1 g/L). Specifically, carnosine was found to restore levels of erythropoietin in the kidney. The beneficial effects of the treatments can be explained by the ability of both compounds to remove hydroxyl radicals and superoxide anions. These antioxidant properties of histidine and carnosine and their positive effect against oxidative stress have been previously highlighted, especially in the context of iron-induced stress and high plasma glucose levels (Vera-Aviles et al. 2018; Gao et al. 2024). In other studies, carnosine supplementation increased carnosine levels in the kidney, reduced albuminuria, mesangial expansion, glomerular basement membrane thickening, and podocyte effacement (Wang et al. 2023a, b). Thus, histidine and carnosine treatments offer protection against oxidative stress in our induced-adenine CKD model, conferring the potential to improve pathological conditions associated with CKD (Kumral et al. 2015; Caruso et al. 2019; Kilis-Pstrusinska 2020).

Carnosine treatment has proved to bind glycation products, such as acrolein and form a stable complex, L-carnosine-acrolein. Subsequently, this complex is eliminated in the urine, diminishing the negative effect of oxidative stress (Menini et al. 2019). Notably, patients with CKD typically suffer from undernutrition or an imbalance of essential amino acids. Thus, the effect of amino acid or peptide supplementation could be improved nutritional status and may not be specific to the effects of histidine or carnosine. Oral or intravenous supplementation of amino acids (with histidine and BCAA amino acids) in uraemia patients with peritoneal dialysis is associated with a beneficial effect on nitrogen metabolism (Divino Filho et al. 1997; Watanabe et al. 2008). The benefits of histidine and carnosine supplementation for CKD-related anaemia and iron supplementation arise from their ability to chelate metals, impact the metabolic pathways involved in ferroptosis, and protect against oxidative stress (Vera-Aviles et al. 2018; Boakye et al. 2019; Caruso et al. 2020, 2022; H. Wang et al. 2023a, b). In models of reperfusion injury and ferroptosis, carnosine protected kidney tissues, reduced MDA levels, iron, but increased the amount of glutathione peroxidase, GSH and superoxide dismutase (Wang et al. 2023a, b).

### Effect of histidine and carnosine in the presence of iron supplementation

Our results also demonstrated that histidine and carnosine co-treated with iron supplementation had a better effect on haemoglobin repletion, ferritin, hepcidin and non-haem iron levels in anaemic CKD model. The increase in non-haem iron is expected within the groups supplemented with FCM, as well as the increase in hepcidin and ferritin in response to increased iron levels. However, it is relevant that histidine and carnosine presented higher haemoglobin repletion compared with iron supplemented only group and with higher levels of non-haem in the liver and ferritin in plasma. These results suggested that histidine and carnosine have a positive effect in intracellular and systemic iron bioavailability, possibly explained by their iron-chelation properties.

Carnosine chelation properties have been shown to modulate the expression of HIF-1α, a crucial regulator of cellular responses to low oxygen levels and iron metabolism. (Forsberg et al. 2015). HIF-1α has been demonstrated to increase the expression levels of iron transporters DMT1 and Tfr1 (Koury and Haase 2015; Locatelli et al. 2017). Carnosine, rather than verapamil (a calcium ion flux modulator), resulted in a higher expression of HIF-1α in H9c2 cardiac cells (Bharadwaj et al. 2002). A comparison of the effect of carnosine with its non-chelating analogue, methylcarcinine, revealed that only carnosine treatment enhanced HIF-1α expression, attributing this effect to carnosine’s metal-binding properties. (Boakye et al. 2019). The metal chelation properties of carnosine have been reported in the context of zinc (Lenz and Martell 1964; Halliwell and Gutteridge 2001). However, considering the histidine affinity constant for iron (logK1(Fe2+) = 5.85, it is likely that both histidine and carnosine form complexes with Fe(II) at physiological pH values (Hider et al. 2021). These are, attesting to carnosine’s ability to chelate and mop up free labile iron, thereby averting cellular ROS generation and improving intracellular and systemic iron availability.

### Effect of histidine and carnosine in iron-overload

It is important to examine the impact of carnosine and histidine treatment on high iron levels or iron overload conditions. Iron-induced overload has been associated with glomerulosclerosis, tubular atrophy, interstitial fibrosis, and iron deposition in the glomeruli, and the proximal and distal tubules (Galleano and Puntarulo 1994; Zhang et al. 2011). In the current study, both histidine and carnosine treatments were found to decrease non-haem iron levels and MDA lipid peroxidation products in kidney tissues. The data suggest that histidine and carnosine treatments mitigate tissue damage, supporting their iron-chelation properties previously observed in anaemic conditions. Furthermore, carnosine demonstrated protective effects against iron overload in both the spleen and kidney, while also increasing total glutathione (GSH) levels in the liver. The increase in GSH levels as a result of carnosine treatment aligns with the proposition that histidine and carnosine could form a stable ternary complex with Fe^2+^ and GSH. In a simulation experiment of glutathione (2 mM) and Fe, along with carnosine (10 mM) at neutral pH, a ternary complex is predicted to form in addition to the ‘GSH-Fe(II) complex’ (Hider et al. 2021). The presence of carnosine influences the cytosolic level of iron(II) glutathione and, in principle, could influence the flow of intracellular Fe^2+^ to major targets, such as the mitochondria. Exploring the role of histidine and carnosine forming ternary complexes with iron(II)glutathione needs further investigation to explain the mechanism underlying the beneficial effects observed in this study.

## Conclusion

Our findings provide evidence that histidine and carnosine enhanced Hb repletion in adenine-induced anaemia of CKD in mice. This is possibly associated with the alleviation of kidney damage by histidine and carnosine, as indicated by reduced BUN levels. Furthermore, both histidine and carnosine decreased non-haem iron and lipid peroxidation in the kidney of iron overload mice. The effectiveness of carnosine is possibly due to the scavenging activity of the imidazole nucleus, which confers antioxidant properties. Co-supplementation of histidine and carnosine with iron could present a synergistic strategy in treating iron deficiency of CKD while neutralizing the resultant toxicity of excess iron. Carnosine has proven beneficial effects in the treatment of oxidative stress and conditions related to iron dysfunction such as anaemia of chronic kidney disease.

## Data Availability

No datasets were generated or analysed during the current study.
